# The 5 kDa Protein NdhP Is Essential for Stable NDH-1L Assembly in *Thermosynechococcus elongatus*

**DOI:** 10.1371/journal.pone.0103584

**Published:** 2014-08-13

**Authors:** Hannes Wulfhorst, Linda E. Franken, Thomas Wessinghage, Egbert J. Boekema, Marc M. Nowaczyk

**Affiliations:** 1 Department of Plant Biochemistry, Ruhr-University Bochum, Bochum, Germany; 2 Electron Microscopy Department, University of Groningen, Groningen, The Netherlands; Arizona State University, United States of America

## Abstract

The cyanobacterial NADPH:plastoquinone oxidoreductase complex (NDH-1), that is related to Complex I of eubacteria and mitochondria, plays a pivotal role in respiration as well as in cyclic electron transfer (CET) around PSI and is involved in a unique carbon concentration mechanism (CCM). Despite many achievements in the past, the complex protein composition and the specific function of many subunits of the different NDH-1 species remain elusive. We have recently discovered in a NDH-1 preparation from *Thermosynechococcus elongatus* two novel single transmembrane peptides (NdhP, NdhQ) with molecular weights below 5 kDa. Here we show that NdhP is a unique component of the ∼450 kDa NDH-1L complex, that is involved in respiration and CET at high CO_2_ concentration, and not detectable in the NDH-1MS and NDH-1MS' complexes that play a role in carbon concentration. C-terminal fusion of NdhP with his-tagged superfolder GFP and the subsequent analysis of the purified complex by electron microscopy and single particle averaging revealed its localization in the NDH-1L specific distal unit of the NDH-1 complex, that is formed by the subunits NdhD1 and NdhF1. Moreover, NdhP is essential for NDH-1L formation, as this type of NDH-1 was not detectable in a Δ*ndhP*::Km mutant.

## Introduction

The cyanobacterial and chloroplast type I NADPH dehydrogenase (NDH-1) complex is structurally and functionally related to the energy-converting NAD(P)H:Quinone oxidoreductase (Complex I) – one key-enzyme of the energy metabolism in eubacteria (like *Escherichia coli*) and the respiratory chain of mitochondria [1]–[3]. This large membrane protein complex is composed of up to ∼45 subunits in mammals with a total molecular weight approaching 1 MDa [4]. Remarkable improvements in the X-ray structural analysis of eubacterial Complex I revealed detailed insights into the electron transfer dependent proton-pumping mechanism on the molecular level [5], [6].

Cyanobacterial NDH-1 and the closely related chloroplast NDH complex are located in the thylakoid membrane and play a pivotal role in respiration (chlororespiration in chloroplasts) as well as in cyclic electron transfer (CET) around PSI [7], [8]. They share the so called oxygenic photosynthesis-specific domain of unknown function, which is composed of NdhL, -M, -N and -O [9] and the basic subunits NdhA-NdhK, which are homologous to subunits of the eubacterial complex. Chloroplast NDH has a more intricate structure and includes several additional proteins compared to cyanobacteria [10]. Most strikingly, the cyanobacterial and chloroplast type NDH-1 complex seem to lack homologues to NuoE/F/G of the eubacterial complex that are responsible for NADH oxidation. This led to the recent idea that electron transfer via ferredoxin might be a possible pathway to chloroplast NDH and cyanobacterial NDH-1 [11], [12].

Based on reverse genetics and proteomic studies, significant progress has been made in resolving the subunit composition and function of the cyanobacterial complex [13], [14] At least four different NDH-1 subtypes (NDH-1L, NDH-1L', NDH-1MS, NDH-1MS') have been identified or postulated for cyanobacteria so far. NDH-1L and NDH-1L' are involved in respiration whereas NDH1-MS and NDH-1MS' link the Complex I specific and electron flow dependent proton-pumping activity to unique carbon concentration mechanisms (CCM) in an unknown process. All NDH-1 variations have been shown to play a role in CET, in particular under stress conditions [15]. They all share a common NDH-1M core unit but they differ in the presence of subunits that are unique for each complex type like NdhF1/D1 (NDH-1L), NdhF1/D2 (NDH-1L'), NdhF3/D3/CupA/CupS (NDH-1MS) and NdhF4/D4/CupB (NDH-1MS'). Two more isoforms of the NdhD subunit (NdhD5, NdhD6) were identified on genome level but they have not been assigned to specific NDH-1 complexes yet.

Although reverse genetic and proteomic studies have already revealed a detailed picture of cyanobacterial NDH-1 complexes, it is still incomplete in many aspects. We have recently identified two novel peptides – NdhP and NdhQ – in purified NDH-1 complexes from *T. elongatus* [16]. NdhP plays a role in NDH-1 mediated electron flow in *Synechocystis* sp. PCC 6803 [17] and shares a weak similarity with NDF6, which is important for NDH-1 activity in *Arabidopsis thaliana* [18]. Here we show by isolation and structural characterization of individual NDH-1 complexes from *T. elongatus* that the 5 kDa NdhP peptide is a unique component of the NDH-1L subtype and essential for its assembly.

## Materials and Methods

### Construction of *T. elongatus* mutants

To delete the *ndhP* gene (genomic region: 1189596–1189465) the coding region was replaced by a kanamycin resistance cassette. Genomic DNA from *T. elongatus* was used as a template for the amplification of the upstream region of *ndhP* by PCR using the specific oligonucleotide primers *ndhP_up_for* and *ndhP_up_KO_rev* (Table S1). The *ndhP* downstream region was amplified using the primer pair *ndhP_down_for* and *ndhP_down_rev*. The DNA fragments were restricted with SacI and XbaI (upstream) and PstI and XhoI (downstream) and ligated into the vector pBluescript SK (+) (Stratagene). A kanamycin resistance cassette was introduced via the XbaI and PstI restriction sites and the resulting plasmid pNdhP_KO was used for transformation of *T. elongatus* according to [19]. Complete segregation of the mutant allele was confirmed by PCR with the primers *SegCheck NdhP_for* and *SegCheck NdhP_rev*.

The NdhP-sfGFP-His_6_ mutant was generated based on a modified pNdhP_KO plasmid. *NdhP* and the corresponding upstream region were replaced by a PCR product (primers: *ndhP_up_for* and *ndhP_up_sfGFP_rev*) that introduces an EcoRV site upstream of the *ndhP* stop codon. The coding region for superfolder GFP [20] with a c-terminal His-Tag (sfGFP-His_6_) was cloned as a synthetic DNA construct (Life Technologies) and introduced into the final plasmid pNdhP-sfGFP-His_6_ via the EcoRV restriction site. After transformation of *T. elongatus* with pNdhP-sfGFP-His_6_, segregation was checked by PCR (primers: *SegCheck NdhP_for* and *SegCheck NdhP_rev*).

To generate the NdhL-TS (TwinStrep-tag) mutant, *ndhL* (*tsr0706*) and the corresponding upstream region were amplified by PCR (primers: *ndhL_up_strep_for* and *OneSTrEP_rev1*.) and the product was used as template for a second PCR (primers: *ndhL_up_strep_for* and *OneSTrEP_XbaI_rev2*). The fragment was cloned into pBluescript SK (+) via SacI and XbaI restriction sites. The *ndhL* downstream region was amplified by PCR (primers: *ndhL_down_for* and *ndhL_down_rev*) and cloned via EcoRI and ApaI. Finally, the kanamycin resistance cassette was introduced via XbaI and EcoRI and the resulting plasmid was used for transformation of *T. elongatus*. Segregation was checked via PCR (primers: *SegCheck_NdhL-strep_for* and *SegCheck_NdhL-strep_rev*).

### Culture conditions – *T. elongatus*

*T. elongatus* wild type strain and mutants (NdhP-sfGFP-His6, Δ*ndhP::Km* and NdhL-TS) were grown in BG-11 liquid medium [21] at 45°C, bubbled with 5% CO_2_ under illumination of increasing intensity of 50–200 µmol photons (dependent on cell density). The medium used for the cultivation of the mutants was supplemented with 80 µg/ml kanamycin.

### Cell harvest and solubilization of thylakoid membranes

The cells were harvested and thylakoids were prepared as described earlier [22]. The buffer composition for n-dodecyl-beta-D-maltoside (DDM, Glycon Biochemicals) solubilization of thylakoid membranes depended on the following purification method. For subsequent Ni^2+^ affinity chromatography the thylakoids were suspended in MES-Buffer (20 mM MES pH 6.5; 25 mM MgCl_2_; 1% (w/v) DDM; 1 mM 4-(2-Aminoethyl) benzenesulfonyl fluoride) and for streptavidin affinity chromatography Tris-buffer (100 mM Tris pH 8.0; 25 mM MgCl_2_; 1% (w/v) DDM; 1 mM 4-(2-Aminoethyl) benzenesulfonyl fluoride) was used. The thylakoid membranes were suspended in the respective buffer at a final chlorophyll concentration of 1 mg/ml and incubated under gentle agitation at 20°C for 60 min. To adjust the final DDM concentration to 0.5% an equal volume of dilution buffer (same composition but without DDM) was added. Insoluble material was removed by centrifugation at 45,000 g.

### Purification of NDH-1 complexes

Solubilized thylakoid membranes were filtered through a 0.45 µm membrane filter and samples containing His-tagged NDH-1 complexes were applied to a 5 ml FF crude IMAC column (GE healthcare) at a flow rate of 1 ml/min after equilibration with IMAC-equilibration buffer (20 mM MES pH 6.5; 0.5 M mannitol; 150 mM NaCl; 0.03% (w/v) DDM). The column was washed with 5 column volumes (CV) IMAC-equilibration buffer containing 10 mM imidazole and His-tagged complexes were eluted with a 10–500 mM imidazole gradient (4 CV) in IMAC-equilibration buffer.

Samples containing TwinStrep-tagged NDH-1 complexes were applied to a 1 ml StrepTactin high capacity column (IBA Biotechnologies) at a flow rate of 1 ml/min after equilibration with Strep-equilibration buffer (100 mM Tris pH 8.0; 0,5 M mannitol; 150 mM NaCl; 0.03% (w/v) DDM). Unbound material was removed by washing with 7 CV Strep-equilibration buffer. Strep-tagged protein complexes were eluted with buffer containing 2.5 mM desthiobiotin. The eluted proteins were concentrated using a spin concentrator with 100 kDa cut off (Millipore) and stored at −80°C.

### Electrophoresis

Blue native (BN)-PAGE was performed according to [23]. Purified proteins (5–10 µg) were mixed with 1/10 volume of sample buffer, loaded on a blue native gradient gel (5–12.5% acrylamid) and electrophoresis was carried out at 4°C at increasing voltage (50–200 V). For electrophoresis in the second dimension, the respective BN gel-lane was cut out, incubated in buffer containing 5% β-mercaptoethanol and 6 M urea and loaded on a 1 mm-thick 14% SDS-PAGE gel with 6 M urea. After electrophoresis (4°C, 13 mA) the proteins were visualized by silver staining according to [24].

### Identification of proteins by 1D-nLC-ESI-MS/MS and MALDI-ToF MS

Protein spots from BN-gels and silver-stained 2D-gels were excised and digested with trypsin as described earlier [16]. The digests were desalted by ZipTips (Millipore), resuspended in 0.1% formic acid in water and analyzed by 1D-nLC-ESI-MS/MS as described in [16]. Intact NDH-1 subunits were examined by MALDI-ToF MS according to [25].

### Transmission electron microscopy and single particle analysis

Purified samples were prepared for negative staining with 2% uranyl acetate on glow-discharged carbon-coated quantifoil grids. Electron microscopy was performed on a Tecnai G2 20 Twin electron microscope (FEI, Eindhoven, the Netherlands) equipped with a LaB_6_ cathode, operated at 200 kV. Images were recorded at 330 nm defocus with an UltraScan 4000 UHS CCD camera (Gatan, Pleasanton, CA, USA) at 100,000-fold nominal magnification with a pixel size of 0.224 nm at the specimen level. GRACE software [26] was used for semi-automated data acquisition. Particle picking was done manually in Eman2 [27]. Output particles were generated in image format and imported into Groningen Image Processing software (GRIP), which was used for further analysis.

A total of 9000 wild type and 15000 NdhP-GFP-labeled NDH1 complexes were collected and processed using standard procedures [28]. Images were pretreated using a low frequency cut-off filter based on the maximum size of the particle and a high frequency cut-off filter based on the maximum resolution available in negative stain (10 Å). The presented images were optimized by application of conditional summing with the correlation coefficient of the final alignment step as a quality parameter to select the most homogeneous images in each class (correlation decay was between 0.1 and 0.2, depending on the number of particles in the class). Resolution of the final results was determined with a Fourier-ring-correlation with a 3 σ threshold criterion.

Comparison of the X-ray structure of NDH-1 from *T. thermophiles* [6] (PDB code: 4HEA) to the electron density map from NDH-1L that was retrieved in this study was done by using UCSF Chimera [29] and Adobe Photoshop. All proteins were fitted separately.

### Bioinformatic tools

Sequence alignment was performed using the Clustal algorithm in the program JalView [30], [31], visualized with the ClustalX residue color code.

Modelling of cyanobacterial subunits was performed using the SwissModel server (http://swissmodel.expasy.org, [32]) with the structure of NDH-1 from *T. thermophilus* [6] (PDB: 4HEA) as template.

## Results and Discussion

### NdhP is a unique component of the NDH-1L complex

We have shown previously that the single-transmembrane protein NdhP co-purifies with NDH-1L complexes, which were isolated via the histidine-rich region of NdhF1 [16] but it remained unclear whether NdhP is also a component of the NDH-1MS or NDH-1MS' complex and the corresponding subcomplexes NDH-1S or NDH-1S'. To answer this question a NdhL-TwinStrep-tag (NdhL-TS) mutant was constructed that enables purification of the other NDH-1 variants. The NdhL subunit is part of the basic NDH-1 unit called NDH-1M and it was shown by Zhang et al. that purification of NDH-1 complexes via NdhL-His resulted in a mixture of NDH-1M, NDH-1L, NDH-1MS and NDH-1S complexes [33]. We used the Strep-tag/Streptactin affinity chromatography system due to its superior purity compared to His-tag affinity chromatography [34].

**Figure 1:**
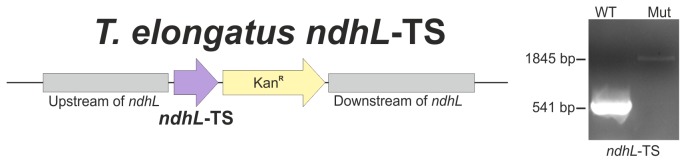
Construction of *T. elongatus* NdhL-TS and segregation check. Schematic representation of the *ndhL* mutant locus (left). The Twin-StrepTag was fused to the NdhL c-terminus and a kanamycin resistance marker was used to force segregation of the mutant allele. Complete segregation was confirmed by PCR analysis (right).

**Figure 2:**
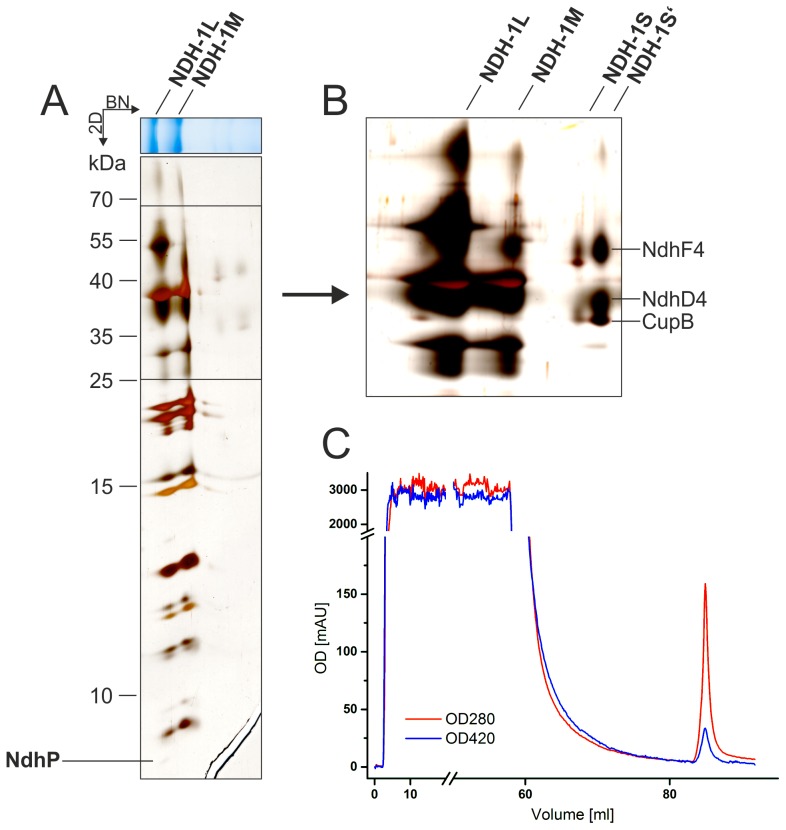
Isolation of NDH-1 complexes via NdhL-TwinStrepTag and 2D-PAGE analysis of purified samples. A: 2D-PAGE analysis isolated NDH-1 complexes. The identity of NDH-1L and NDH-1M as well as NdhP was confirmed by MS/MS analysis (see Table S2, S3 and S6). B: Identification of NDH-1S and NDH-1S' by 2D-PAGE analysis and mass spectrometry (see Table S4 and S5). C: Tagged NDH-1 complexes were purified by StrepTactin affinity chromatography.

The TwinStrep-tag was fused to the c-terminus of NdhL and the complete segregation of the NdhL-TS mutant was confirmed by PCR analysis (Fig. 1). Solubilized membranes of 5 L culture were subjected to StrepTactin affinity chromatography and the concentrated elution fractions were analyzed by BN- and 2D-gelelectrophoresis (Fig. 2). Four different NDH-1 complexes were separated by BN-PAGE and the identity of NDH-1L, NDH-1M and NDH-1S was confirmed by high resolution LC-MS/MS (Tables S2, S3 and S4) and MALDI-ToF MS analysis (Fig. S1). Moreover, all specific subunits of NDH-1S' (NdhD4, NdhF4, CupB) were identified by MS analysis (Table S5) which confirms – for the first time to our knowledge – its existence on protein level.

The NdhP subunit was solely detected in the NDH-1L complex by identification of a specific peptide (Table S6, Fig. S2) that was missing in NDH-1M, NDH-1S and NDH-1S' samples. Although no corresponding bands of the intact NDH-1MS or NDH-1MS' complex are visible in the BN-PAGE analysis, NdhP can be assigned to the NDH-1L complex – most likely inside the NdhD1/F1 unit - as it is not present in any of the corresponding subcomplexes.

### Electron microscopy localizes NdhP in the distal NdhD1/F1 unit of NDH-1L

It was previously shown that fusion of several NDH-1 subunits with YFP and the subsequent analysis by electron microscopy and single particle averaging revealed the position of the tagged subunits inside the NDH-1 complex of *Synechocystis* sp. [9]. To investigate the distinct localization of NdhP within the NDH-1L complex, we fused the 27 kDa superfolder GFP (sfGFP) protein [20] with an additional His-tag to the NdhP c-terminus. A c-terminal fusion was applied to ensure cytoplasmic localization of the sfGFP tag, as the n-terminus of the single transmembrane helix protein NdhP was predicted to be oriented towards the lumen [16]. This construct should avoid misfolding as well as impaired membrane insertion of NdhP caused by the sfGFP fusion. The thermophilic target strain *T. elongatus* was cultured at elevated temperature (45°–50°C) for optimal growth. Superfolder GFP was used for the tagging approach, as it shows enhanced thermostability compared to other GFP variants [35].

**Figure 3:**
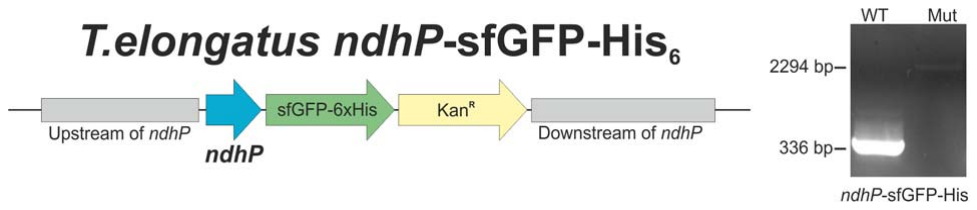
Construction of *T. elongatus* NdhP-sfGFP-His and segregation check. Schematic representation of the *ndhP* mutant locus (left). The sfGFP-His6-Tag was fused to the NdhP c-terminus and a kanamycin resistance marker was used to force segregation of the mutant allele. Complete segregation was confirmed by PCR analysis (right).

**Figure 4:**
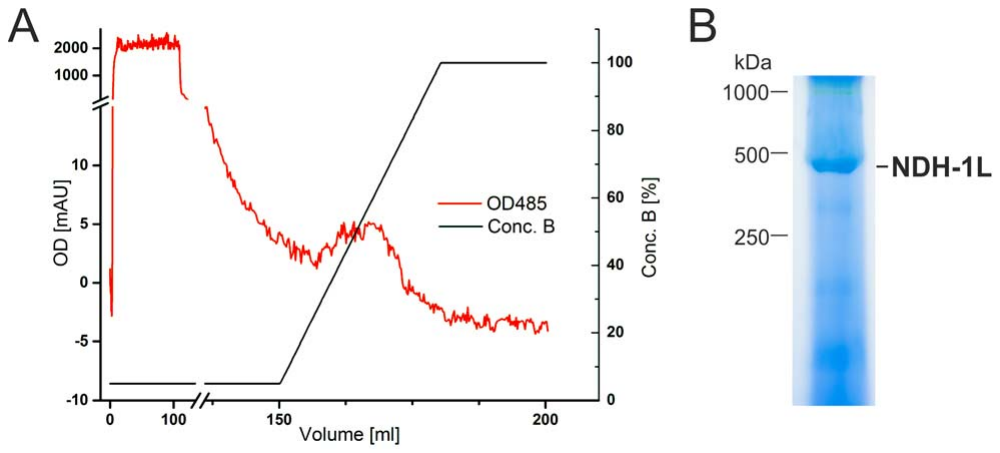
Isolation of NDH-1L via NdhP-sfGFP-His by Ni-affinity chromatography. **A:** His-tagged NDH-1L complexes were eluted from a Ni-NTA column with a linear gradient of 10–500 mM imidazole. The absorption of sfGFP at 485 nm was used to follow the purification. B: BN-PAGE of eluted proteins. The band with an apparent molecular weight of approximately 450 kDa was identified as NDH-1L by MS/MS analysis (see Table S7 and S8).

The complete segregation of the *T. elongatus* NdhP-sfGFP-His mutant was confirmed by PCR analysis (Fig. 3). His-sfGFP-tagged NDH-1 complexes were isolated via Ni-affinity chromatography (Fig. 4A) and analyzed by BN-PAGE (Fig. 4B). As expected, only a single protein complex with a molecular weight of approximately 450 kDa was isolated and mass spectrometry analysis revealed the presence of the NDH-1L specific subunits NdhD1 and NdhF1 (Table S7). The NdhP-sfGFP-fusion was verified by mass spectrometry, as a specific peptide of the linker region that connects NdhP and sfGFP, was identified in the sample (Table S8, Fig. S3).

**Figure 5:**
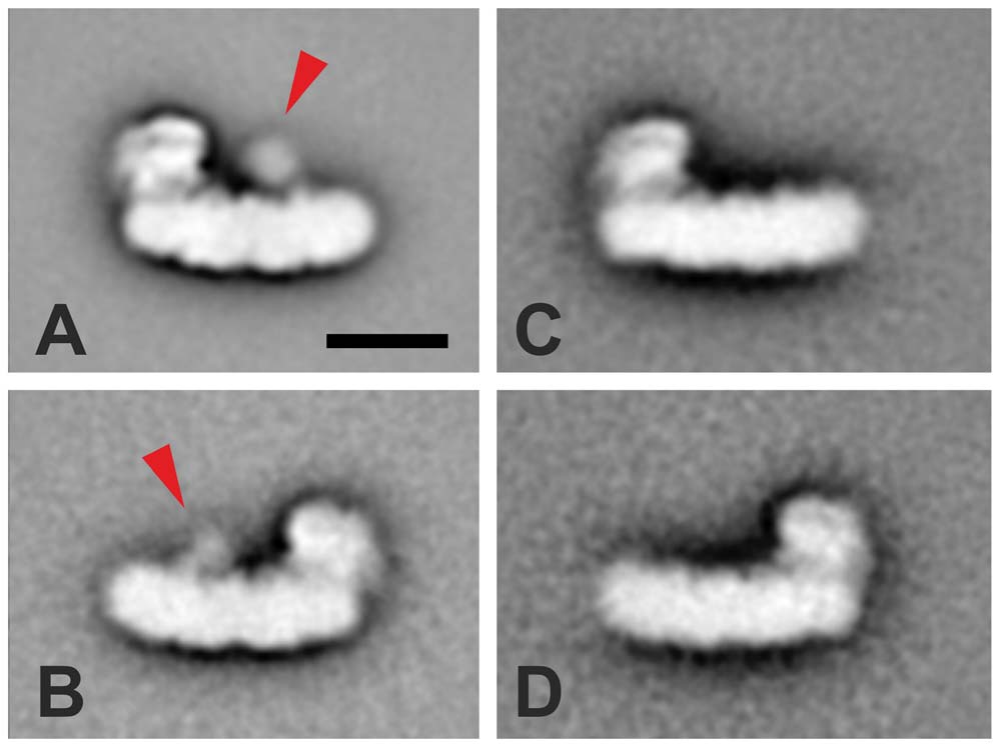
Projection maps of NdhP-sfGFP-His-tagged (A and B) and NdhL-TS-tagged NDH-1L complexes (C and D). A is a conditional sum of 9158 images (correlation decay: 0.2) at 16 Å resolution. B contains 1326 images (corr. d.: 0.15) at 25 Å resolution. Projection map C is the sum of 4409 images (corr. d.: 0.15) at 26 Å. D sums 1758 images (corr. d. 0.2) to a 27 Å resolution map. The position of the sfGFP-tag is indicated by red arrows. The scale bar represents 10 nm.

Affinity purified NDH-1 complexes from both *T. elongatus* NdhP-sfGFP-His and NdhL-TS mutant were studied by electron microscopy. A total of 15000 NdhP-sfGFP-labeled and 9000 NdhL-TS-labeled NDH-1L complexes were collected (see Materials and Methods) and the selected single particle projections were analyzed by single particle averaging and assigned to homogenous classes, followed by subsequent averaging of class members into 2D maps. From NDH-1L of *T. elongatus* NdhP-sfGFP-His two classes of differently oriented complexes were generated, resulting in a left- and right-handed side view (Fig. 5A+B). Compared to the projection maps of NDH-1L of *T. elongatus* NdhL-TS (Fig. 5C+D) an extra density is visible in both side views of NDH-1L from NdhP-sfGFP-His (red arrows), which has to be the sfGFP-His-tag. In conclusion, the previously predicted orientation of NdhP [16] is clearly confirmed by the results of the structural analysis. Based on the asymmetric shape of NDH-1L, one can assign the c-terminally fused sfGFP-tag to the cytoplasmic side of the membrane.

Moreover, the size of the tag is optically different between side view A and B. This is best explained by the tag pointing towards the viewer out of the stain-layer in side view A and away from the viewer into the stain-layer in side view B. This indicates that the tag is not exactly in the middle.

**Figure 6:**
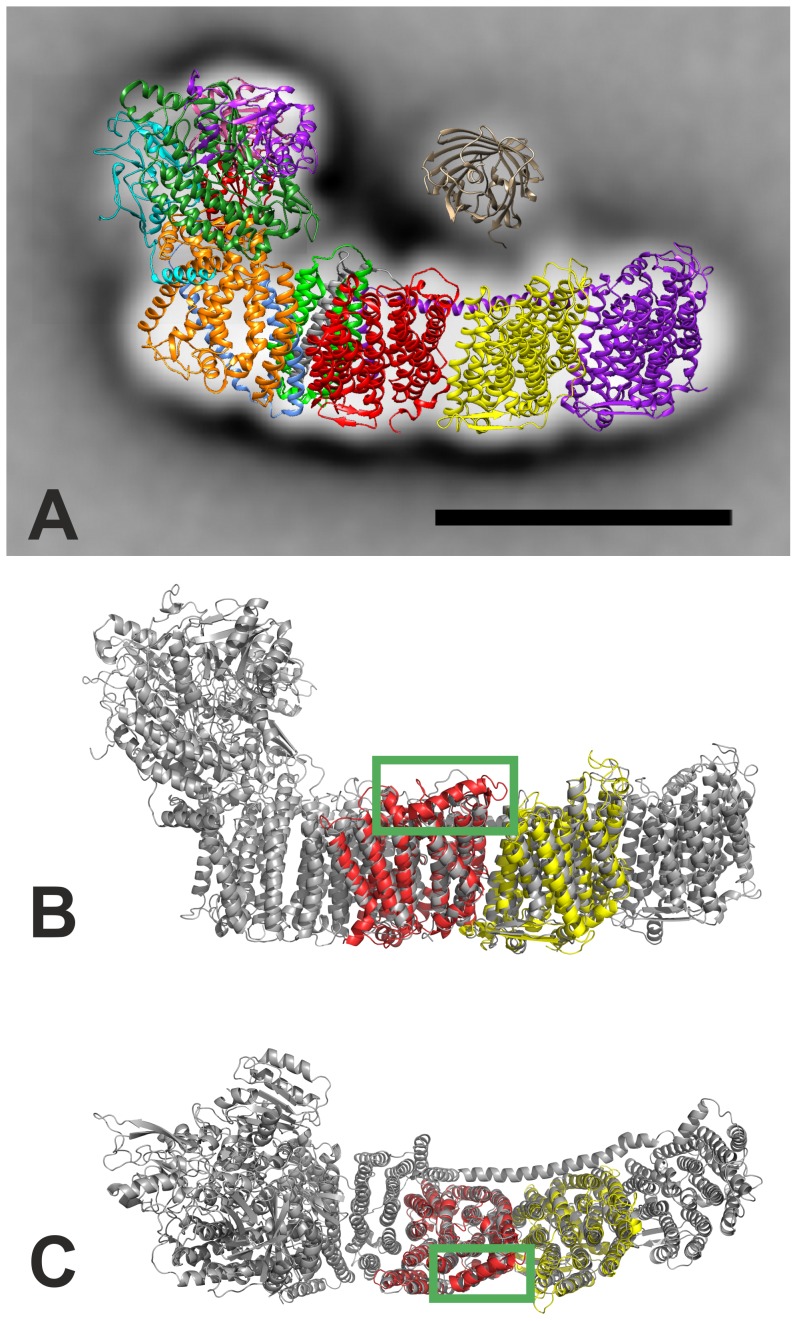
Localization of the NdhP subunit. A: Fitting of the NDH-1L projection map with sfGFP [20] (PDB code: 2B3P) to the X-ray structure of NDH-1 from *T. thermophilus* [6] (PDB code: 4HEA). The scale bar represents 10 nm. Colour code: Nqo4 (NdhH): dark green, Nqo5 (NdhJ): light purple, Nqo6 (NdhK): light red, Nqo7 (NdhC): light blue, Nqo8 (NdhA): orange, Nqo9 (NdhI): cyan, Nqo10 (NdhG): green, Nqo11 (NdhE): grey, Nqo12 (NdhF1): purple, Nqo13 (NdhD1): yellow, Nqo14 (NdhB): red. B and C: Structural comparison of the eubacterial NDH-1 complex [6] with tentative models of the NDH-1 subunits NdhB and NdhD1 from *T. elongatus* (B: side view, C: top view). Green square: additional helix in NdhB of *T. elongatus*; NdhB is shown in red and NdhD1 in yellow. The subunits Nqo1-3 from *T. thermophilus* are missing in the cyanobacterial NDH-1 complex and were therefore omitted.

By fitting the 2D projection map with the homologous part of the X-ray structure of NDH-1 from *T. thermophilus* [6] (Fig. 6A) the position of the sfGFP-His-tag could be specified to be “above” the subunits NdhB (Nqo13, red) and NdhD1 (Nqo14, yellow), possibly with its c-terminus pointing more into the direction of NdhD1. Interestingly, the structures of Nqo7 (NdhC), Nqo8 (NdhA) and the hydrophilic domain (Nqo4/NdhH, Nqo9/NdhI, Nqo5/NdhJ, Nqo6/NdhK) had to be rotated to make them fit to the 2D projection map. This changes the direction of these subunits from pointing to the right to pointing away from the viewer. A more detailed structural comparison of the Nqo13 and Nqo14 subunits from eubacterial NDH-1 [6] with models of NdhB and NdhD1 from *T. elongatus* (Fig. 6B, 6C and S4) revealed that the general structure (position of transmembrane helices) seem to be consistent. One striking difference between both complexes might be an additional helix at the cytoplasmic side of NdhB. A tentative localization of NdhP in this part of the complex would be in good agreement with our data.

### Isolation of NDH-1L via NdhF1 is impaired in a Δ*ndhP*::Km mutant

**Figure 7:**
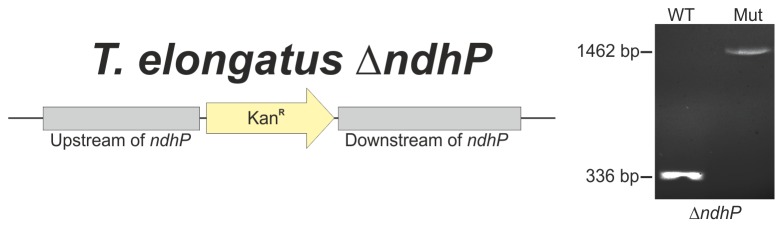
Construction of *T. elongatus* Δ*ndhP* and segregation check. Schematic representation of the Δ*ndhP* mutant locus (left). A kanamycin resistance marker was used to replace the entire *ndhP* gene. Complete segregation was confirmed by PCR analysis (right).

**Figure 8:**
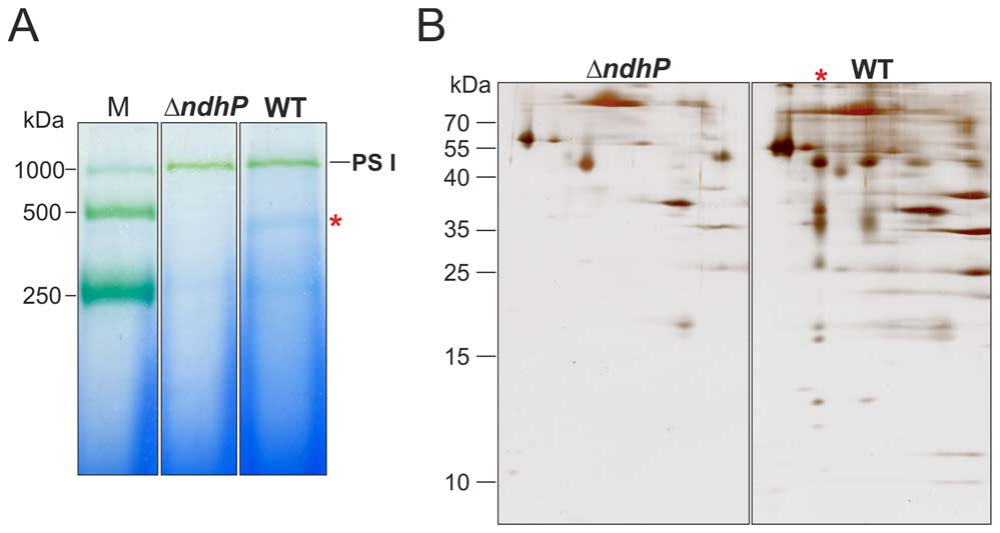
Comparative purification of NDH-1 complexes via NdhF1 from wild type and Δ*ndhP*::Km mutant cells by Ni-affinity chromatography. A: BN-PAGE analysis of purified complexes. The 450 kDa complex in the wild type (WT) lane is indicated by a red asterisk. B: 2D-PAGE analysis. The band pattern of the respective complex (red asterisk) is characteristic for NDH-1L.

To investigate the role of NdhP in the NDH-1L complex, a comparative purification from *T. elongatus* wild type and a Δ*ndhP*::Km mutant was conducted by Ni-affinity chromatography via the histidine rich region of the NdhF1 subunit [33]. The mutant was generated by replacement of *ndhP* with a kanamycin resistance cassette and complete segregation was confirmed by PCR analysis (Fig. 7). Equivalent amounts (10 mg Chl) of thylakoid membranes from wild type and mutant were solubilized and applied to Ni-affinity chromatography. BN-PAGE analysis (Fig. 8A) of purified protein complexes revealed the presence of a putative NDH-1L complex (red asterisk) in the wild type sample that is missing in the Δ*ndhP*::Km mutant. Both samples contain equal amounts of co-eluted trimeric PSI complexes that are typical contaminants for this type of purification [36]. The characteristic protein spot pattern that is visible in the SDS-PAGE dimension (Fig. 8B, red asterisk) confirms the presence of NDH-1L in the wild type sample and its absence in the Δ*ndhP*::Km mutant.

In a recent study Schwarz et al. analyzed a Δ*ndhP::Km* mutant of *Synechocystis* sp. [17]. It showed reduced NDH-1 mediated cyclic electron transport (CET) around PSI under high CO_2_ (HC) conditions, whereas under low CO_2_ (LC) no significant change was observed. Interestingly, NDH-1L is the dominant complex under HC conditions, whereas NDH-1MS is strongly induced under LC conditions. Since the latter complex also contributes to CET, the authors speculate that NdhP is a NDH-1L specific subunit that mediates efficient CET under HC conditions. Here we can clearly show that NdhP is a unique and essential component of the NDH-1L complex. NDH-1L is not detectable anymore in the Δ*ndhP*::Km mutant and - as a direct consequence - this mutant shows the same phenotype [17] as Δ*ndhD1* and Δ*ndhF1*, which also lack the NDH-1L complex [15], [23].

**Figure 9:**
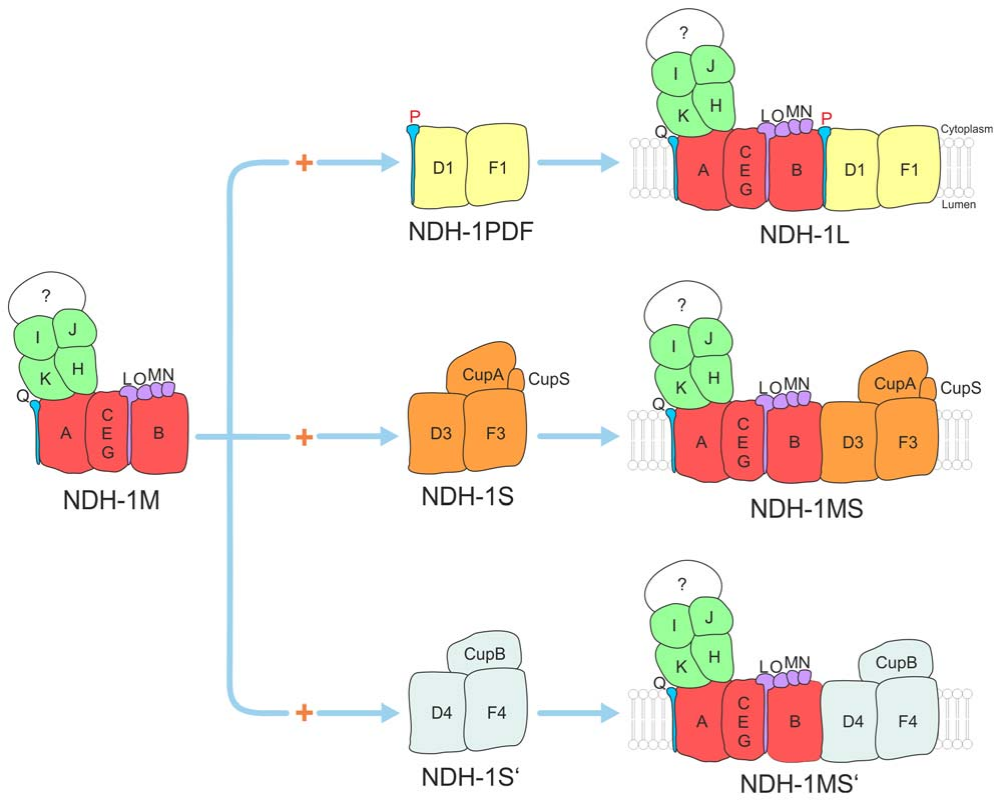
Model of NDH-1 complexes that have been verified on protein level. The basic complex NDH-1M is combined with specific domains to assemble the functional complexes NDH-1L, NDH-1MS' and NDH-1MS.

In conclusion, the present study corroborated the important role of NdhP within the cyanobacterial NDH-1 complex. NdhP is located in the distal unit of NDH-1 that is formed by the NdhB and NdhD1 subunits. It was allocated specifically to the NDH-1L complex, which is involved in respiration and CET under high CO_2_ concentration (Fig. 9). And obviously NdhP is essential for the stable assembly of this multisubunit membrane protein complex, as it is not detectable anymore in the Δ*ndhP*::Km mutant. A similar observation was published very recently for the NDH-1L complex of *Synechocystis* sp. PCC 6803 [37]. Although, they are challenging to identify and sometimes overlooked, single-transmembrane domain proteins seem to play a central role in the organization of membrane protein complexes in general [38] and the 5 kDa NdhP single-transmembrane NDH-1 subunit is one striking example for this often undervalued class of proteins.

## Supporting Information

Figure S1 **MALDI-TOF spectra of NDH-1 preparations from A: NdhL-TS, B: NdhP-sfGFP-His.** NdhQ is present in both preparations, whereas NdhP is detected only in the NdhL-TS preparation due to its additional mass in NdhP-sfGFP-His.
(TIFF)

Figure S2 **Fragment spectrum of NdhP peptide.**
(TIFF)

Figure S3 **Precursor and fragment spectrum of NdhP-sfGFP-His peptide.**
(TIFF)

Figure S4 **Sequence alignment of NdhB.** Red square: position of the additional helix in NdhB of *T. elongatus*.
(TIFF)

Table S1 **Primers used in this study.**
(DOCX)

Table S2 **NDH-1L subunit analysis after in-gel digestion with trypsin.**
(DOCX)

Table S3 **NDH-1M subunit analysis after in-gel digestion with trypsin.**
(DOCX)

Table S4 **NDH-1S subunit analysis after in-gel digestion with trypsin.**
(DOCX)

Table S5 **NDH-1S' subunit analysis after in-gel digestion with trypsin.**
(DOCX)

Table S6 **Identification of NdhP by specific peptide.**
(DOCX)

Table S7 **NDH-1L-sfGFP subunit analysis after in-gel digestion with trypsin.**
(DOCX)

Table S8 **Identification of NdhP-sfGFP-His by specific peptides.**
(DOCX)
