# Supplementary figures and images for: The 5 kDa Protein NdhP Is Essential for Stable NDH-1L Assembly in Thermosynechococcus elongatus
Source: PLoS One. 2014 Aug 13;9(8):e103584. doi: 10.1371/journal.pone.0103584 (PMC4131877; doi:10.1371/journal.pone.0103584)

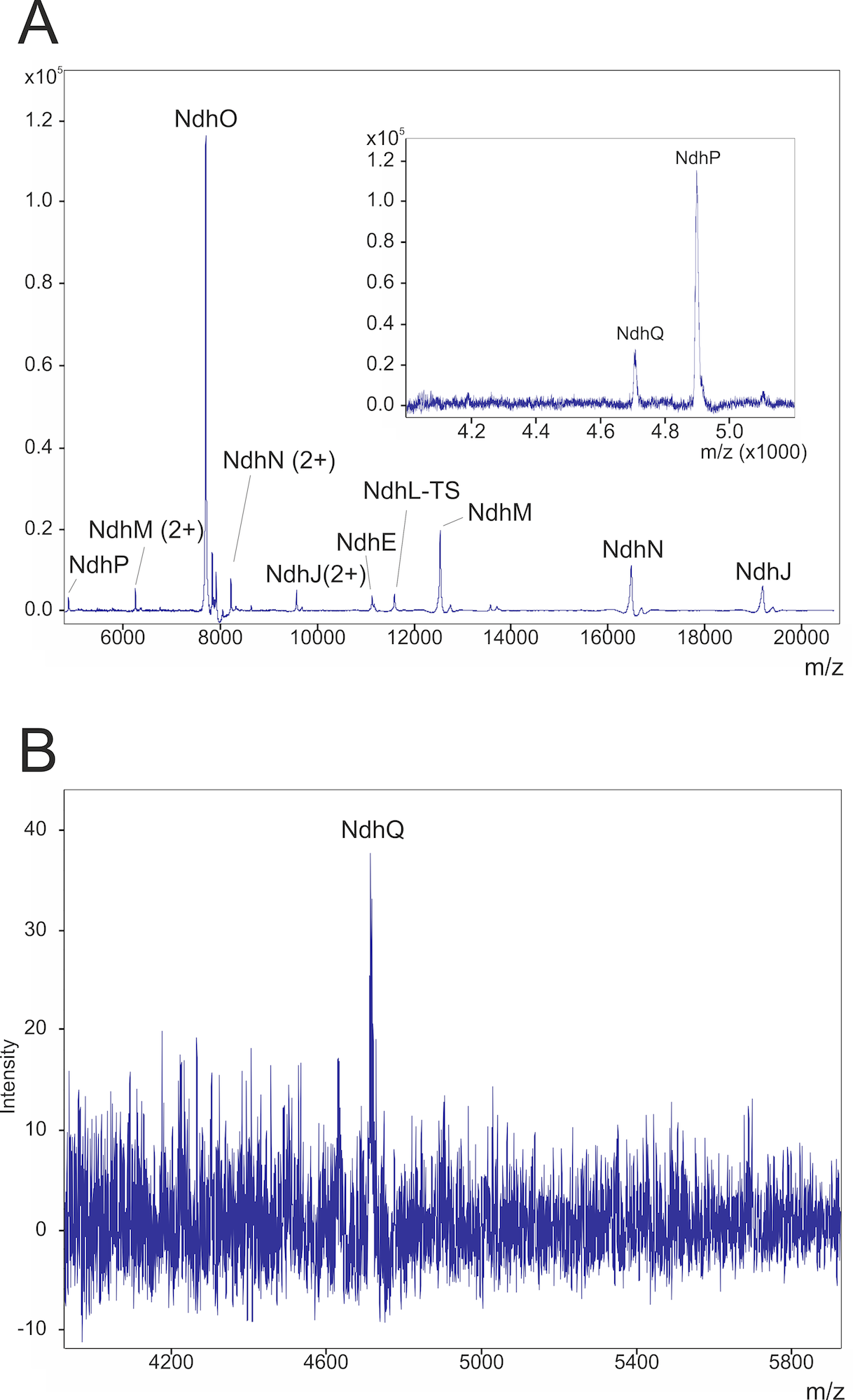

Supplement: Figure S1 — MALDI-TOF spectra of NDH-1 preparations from A: NdhL-TS, B: NdhP-sfGFP-His. NdhQ is present in both preparations, whereas NdhP is detected only in the NdhL-TS preparation due to its additional mass in NdhP-sfGFP-His. (TIFF) [file pone.0103584.s001.tiff]

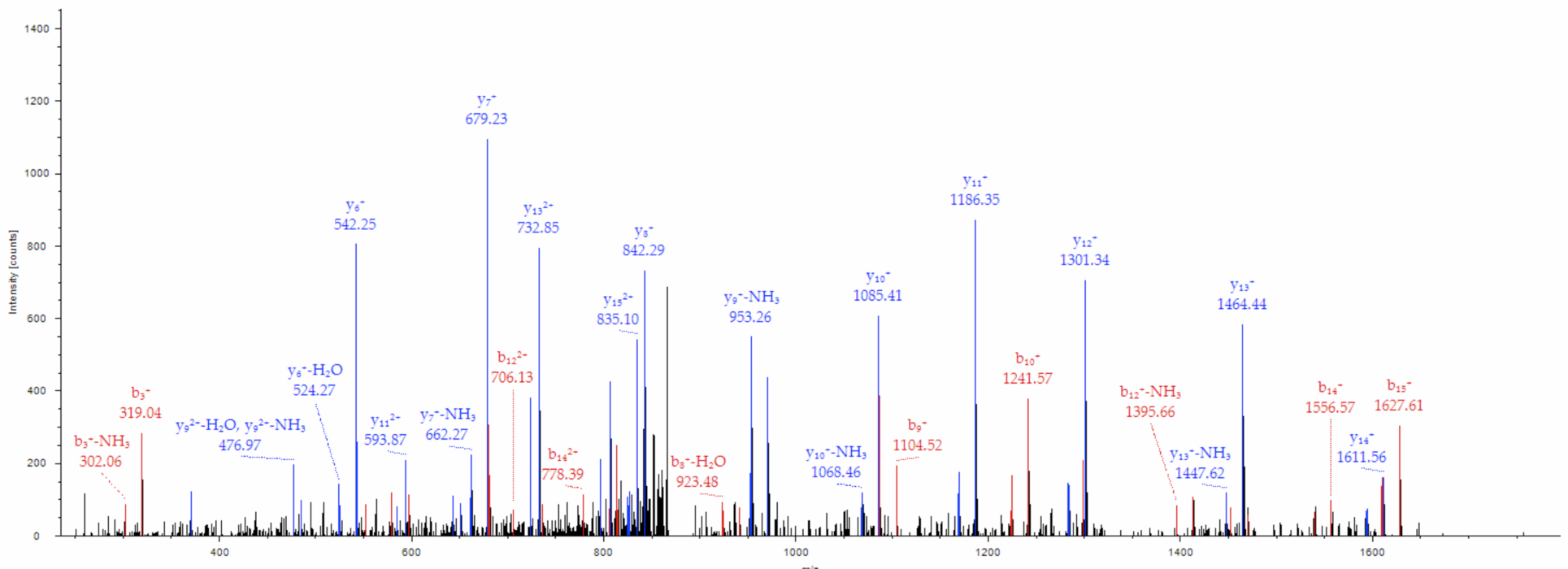

Supplement: Figure S2 — Fragment spectrum of NdhP peptide. (TIFF) [file pone.0103584.s002.tiff]

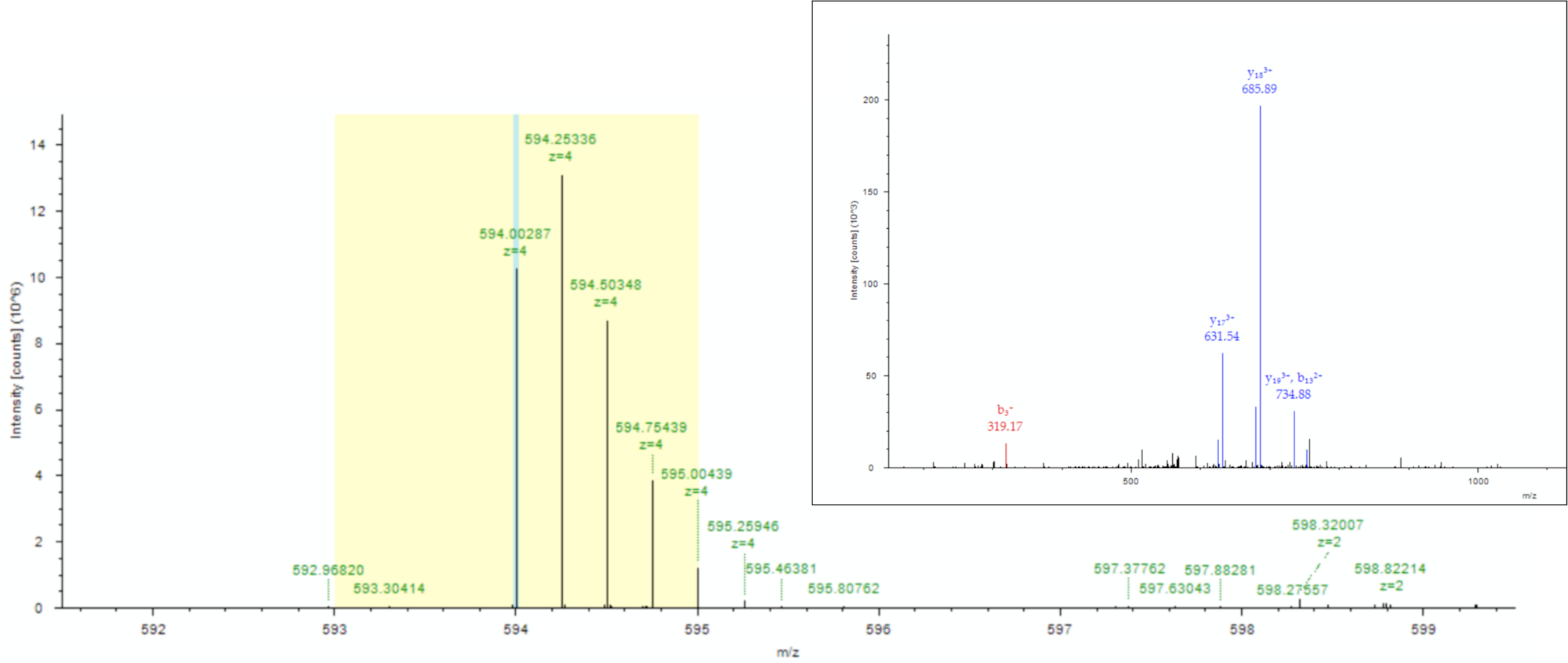

Supplement: Figure S3 — Precursor and fragment spectrum of NdhP-sfGFP-His peptide. (TIFF) [file pone.0103584.s003.tiff]

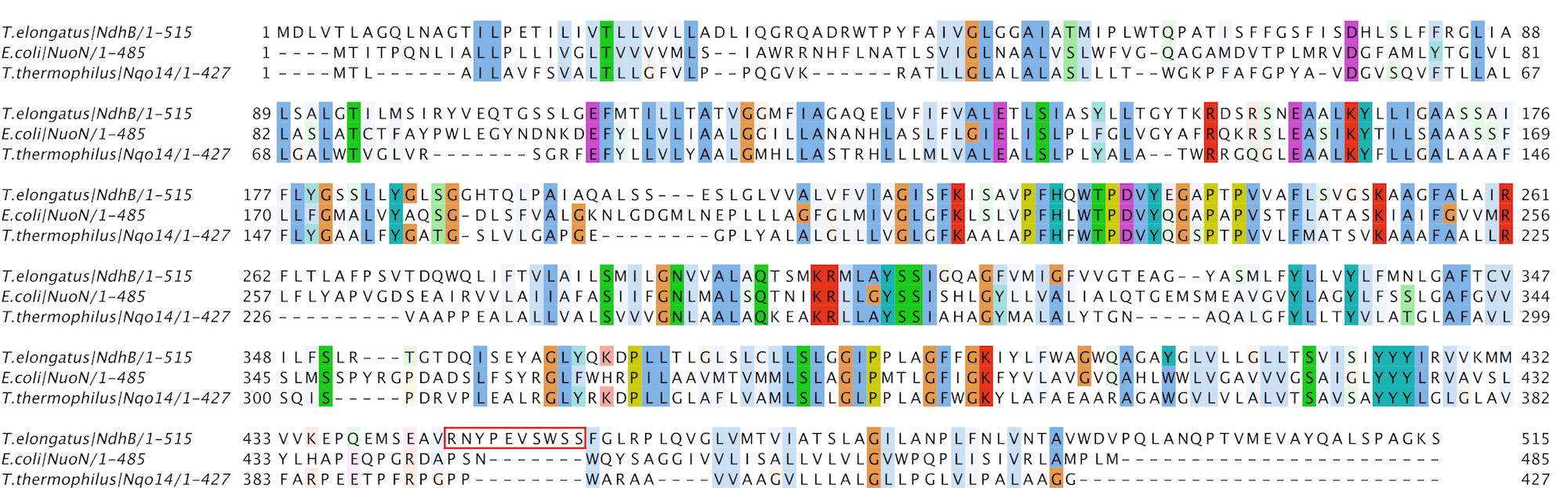

Supplement: Figure S4 — Sequence alignment of NdhB. Red square: position of the additional helix in NdhB of T. elongatus. (TIFF) [file pone.0103584.s004.tiff]
