# Supplementary material for: The 5 kDa Protein NdhP Is Essential for Stable NDH-1L Assembly in Thermosynechococcus elongatus
Source: PLoS One. 2014 Aug 13;9(8):e103584. doi: 10.1371/journal.pone.0103584 (PMC4131877; doi:10.1371/journal.pone.0103584)
Supplement: Table S1 — Primers used in this study. (DOCX) [file pone.0103584.s005.docx]

| Name | Sequence (5’-3’) |
| --- | --- |
|  |  |
| *ndhP_up_for* | AAAGAGCTCACGGACCAATCCAGATGTGT |
| *ndhP_up_KO_rev* | GGGTCTAGAAATACAGGCGTCATAGCCAG |
| *ndhP_down_for* | GGGCTGCAGATCTAACAAGGGAGGGTGAC |
| *ndhP_down_rev* | CCCCTCGAGATAGAAGGCGGTAGGCGAAT |
| *SegCheck NdhP_for* | GACATCAAGAGACGGCACAC |
| *SegCheck NdhP_rev* | CCGCGATACACTGAGTACCA |
| *ndhP_up_sfGFP_rev* | GGCTCTAGAGATATCGTGGGCAGAACCGTTACCGTG |
| *ndhL_up_strep_for* | AAAGAGCTCGTCGGATGTGGGGGCAGCAG |
| *OneSTrEP_rev1* | GCAGAACCACCAGAACCACCGCCGCTGCCGCCGCCTTTTTCGAACTGCGGGTGGC |
| *OneSTrEP_XbaI_rev2* | CCCTCTAGATTACTTCTCAAATTGCGGATGAGACCACGCAGAACCACCAGAACCACC |
| *ndhL_down_for* | GGGGAATTCGTGCTATGCGCCGCATTGAT |
| *ndhL_down_rev* | AAAGGGCCCAAGGCCGCTTGCAAAGCTTC |
| *SegCheck_NdhL-strep_for* | ATCTCAATGCGCCTAGTCGG |
| *SegCheck_NdhL-strep_rev* | TGGAGAGCCAGCCAAGAATC |
