# Supplementary material for: The 5 kDa Protein NdhP Is Essential for Stable NDH-1L Assembly in Thermosynechococcus elongatus
Source: PLoS One. 2014 Aug 13;9(8):e103584. doi: 10.1371/journal.pone.0103584 (PMC4131877; doi:10.1371/journal.pone.0103584)
Supplement: Table S2 — NDH-1L subunit analysis after in-gel digestion with trypsin. (DOCX) [file pone.0103584.s006.docx]

|  | | | | | |  |
| --- | --- | --- | --- | --- | --- | --- |
| NDH-1 SU | ORF | kDa | TMH | XC | Coverage | |
|  |  |  |  |  |  | |
| NdhA | tlr0667 | 41.3 | 13 | 85.16 | 24.80 | |
| NdhB | tll0045 | 55.11 | 14 | 84.60 | 5.83 | |
| NdhC | tlr1429 | 14.99 | 3 | 13.51 | 15.91 | |
| NdhD1 | tll0719 | 56.04 | 12 | 351.28 | 17.90 | |
| NdhE | tlr0670 | 11.13 | 3 | 8.47 | 11.88 | |
| NdhF1 | tll0720 | 71.93 | 16 | 22.93 | 3.20 | |
| NdhG | tlr0669 | 21.56 | 5 | 16.42 | 21.50 | |
| NdhH | tlr1288 | 45.19 |  | 605.33 | 41.88 | |
| NdhI | tlr0668 | 22.40 |  | 242.34 | 54.08 | |
| NdhJ | tlr1430 | 19.33 |  | 190.31 | 71.43 | |
| NdhK | tlr0705 | 25.73 |  | 288.34 | 39.66 | |
| NdhL | tsr0706 | 8.60 | 2 | 12.29 | 11.84 | |
| NdhM | tll0447 | 12.56 |  | 287.57 | 46.85 | |
| NdhO | tsl0017 | 7.86 |  | 22.64 | 54.29 | |
| NdhP |  | 4.90 | 1 | 8.34 | 36.36 | |
| NdhS | tlr0636 | 12.44 |  | 53.89 | 41.82 | |
