# Supplementary material for: The 5 kDa Protein NdhP Is Essential for Stable NDH-1L Assembly in Thermosynechococcus elongatus
Source: PLoS One. 2014 Aug 13;9(8):e103584. doi: 10.1371/journal.pone.0103584 (PMC4131877; doi:10.1371/journal.pone.0103584)
Supplement: Table S3 — NDH-1M subunit analysis after in-gel digestion with trypsin. (DOCX) [file pone.0103584.s007.docx]

| NDH-1 SU | ORF | kDa | TMH | XC | Coverage |
| --- | --- | --- | --- | --- | --- |
|  |  |  |  |  |  |
| NdhA | tlr0667 | 41.3 | 13 | 38.44 | 18.47 |
| NdhB | tll0045 | 55.11 | 14 | 45.00 | 5.83 |
| NdhE | tlr0670 | 11.13 | 3 | 17.78 | 12.87 |
| NdhG | tlr0669 | 21.56 | 5 | 7.74 | 12.50 |
| NdhH | tlr1288 | 45.19 |  | 218.82 | 31.98 |
| NdhI | tlr0668 | 22.40 |  | 211.35 | 47.45 |
| NdhJ | tlr1430 | 19.33 |  | 80.05 | 38.10 |
| NdhK | tlr0705 | 25.73 |  | 138.14 | 26.16 |
| NdhL | tsr0706 | 8.60 | 2 | 2.39 | 11.84 |
| NdhM | tll0447 | 12.56 |  | 19.78 | 37.84 |
| NdhN | tlr1130 | 16.60 |  | 65.33 | 50.67 |
| NdhO | tsl0017 | 7.86 |  | 3.14 | 25.71 |
| NdhS | tlr0636 | 12.44 |  | 24.36 | 30.91 |
