# Supplementary material for: The 5 kDa Protein NdhP Is Essential for Stable NDH-1L Assembly in Thermosynechococcus elongatus
Source: PLoS One. 2014 Aug 13;9(8):e103584. doi: 10.1371/journal.pone.0103584 (PMC4131877; doi:10.1371/journal.pone.0103584)
Supplement: Table S4 — NDH-1S subunit analysis after in-gel digestion with trypsin. (DOCX) [file pone.0103584.s008.docx]

| NDH-1 SU | ORF | kDa | TMH | XC | Coverage |
| --- | --- | --- | --- | --- | --- |
|  |  |  |  |  |  |
| NdhD3 | tlr0905 | 53.87 | 12 | 136.39 | 20.56 |
| NdhF3 | tlr0904 | 66.25 | 16 | 565.90 | 15.55 |
| CupA | tlr0906 | 50.94 |  | 1054.16 | 60.18 |
| CupS | tll0220 | 15.78 |  | 236.17 | 78.52 |
