# Supplementary material for: The 5 kDa Protein NdhP Is Essential for Stable NDH-1L Assembly in Thermosynechococcus elongatus
Source: PLoS One. 2014 Aug 13;9(8):e103584. doi: 10.1371/journal.pone.0103584 (PMC4131877; doi:10.1371/journal.pone.0103584)
Supplement: Table S5 — NDH-1S' subunit analysis after in-gel digestion with trypsin. (DOCX) [file pone.0103584.s009.docx]

| NDH-1 SU | ORF | kDa | TMH | XC (BN) | Coverage (BN) | XC (2D) | Coverage (2D) |
| --- | --- | --- | --- | --- | --- | --- | --- |
|  |  |  |  |  |  |  |  |
| NdhD4 | tlr2125 | 53.38 | 12 | 12.24 | 4.82 | 13.71 | 8.43 |
| NdhF4 | tlr2124 | 66.14 | 16 | 86.27 | 7.55 | 19.36 | 5.75 |
| CupB | tlr2126 | 43.26 |  | 52.53 | 28.68 | 46.70 | 20.26 |
