# Supplementary material for: The 5 kDa Protein NdhP Is Essential for Stable NDH-1L Assembly in Thermosynechococcus elongatus
Source: PLoS One. 2014 Aug 13;9(8):e103584. doi: 10.1371/journal.pone.0103584 (PMC4131877; doi:10.1371/journal.pone.0103584)
Supplement: Table S6 — Identification of NdhP by specific peptide. (DOCX) [file pone.0103584.s010.docx]

| Peptide (Sequence) | MH+ [Da] | Charge | ΔM [ppm] | XCorr |
| --- | --- | --- | --- | --- |
|  |  |  |  |  |
| NGFYDTDQYHGNGSAH | 1782.70854 | 2 | -0.99 | 4.3 |
