# Supplementary material for: The 5 kDa Protein NdhP Is Essential for Stable NDH-1L Assembly in Thermosynechococcus elongatus
Source: PLoS One. 2014 Aug 13;9(8):e103584. doi: 10.1371/journal.pone.0103584 (PMC4131877; doi:10.1371/journal.pone.0103584)
Supplement: Table S7 — NDH-1L-sfGFP subunit analysis after in-gel digestion with trypsin. (DOCX) [file pone.0103584.s011.docx]

| NDH-1 SU | ORF | kDa | TMH | XC | Coverage |
| --- | --- | --- | --- | --- | --- |
|  |  |  |  |  |  |
| NdhA | tlr0667 | 41.3 | 13 | 1327.70 | 22.43 |
| NdhB | tll0045 | 55.1 | 14 | 217.15 | 7.18 |
| NdhD1 | tll0719 | 56.0 | 12 | 737.80 | 20.82 |
| NdhE | tlr0670 | 11.1 | 3 | 30.92 | 13.86 |
| NdhF1 | tll0720 | 71.9 | 16 | 150.65 | 7.01 |
| NdhG | tlr0669 | 21.6 | 5 | 83.27 | 21.50 |
| NdhH | tlr1288 | 45.2 |  | 2782.46 | 56.60 |
| NdhI | tlr0668 | 22.4 |  | 1003.82 | 56.63 |
| NdhJ | tlr1430 | 19.3 |  | 732.08 | 54.76 |
| NdhK | tlr0705 | 25.7 |  | 1080.44 | 43.04 |
| NdhL | tsr0706 | 8.6 | 2 | 11.80 | 11.84 |
| NdhM | tll0447 | 12.6 |  | 231.12 | 45.95 |
| NdhO | tsl0017 | 7.9 |  | 78.69 | 65.71 |
| NdhS | tlr0636 | 12.4 |  | 46.42 | 41.82 |
|  |  |  |  |  |  |
| NdhP-sfGFP-His |  | 32.8 | 1 | 14.43 | 16.44 |
