# Supplementary material for: The 5 kDa Protein NdhP Is Essential for Stable NDH-1L Assembly in Thermosynechococcus elongatus
Source: PLoS One. 2014 Aug 13;9(8):e103584. doi: 10.1371/journal.pone.0103584 (PMC4131877; doi:10.1371/journal.pone.0103584)
Supplement: Table S8 — Identification of NdhP-sfGFP-His by specific peptides. (DOCX) [file pone.0103584.s012.docx]

| Peptide (Sequence) | MH+ [Da] | Charge | ΔM [ppm] | Modifications | XCorr |
| --- | --- | --- | --- | --- | --- |
|  |  |  |  |  |  |
| LEYNFNSHNVYITADK | 1927.927707 | 3 | 4.77 |  | 3.74 |
| NGFYDTDQYHGNGSAHDImSK | 2372.989645 | 4 | 2.49 | M19(Oxidation) | 2.65 |
| SAMPEGYVQER | 1266.582079 | 2 | 2.93 |  | 2.14 |
